# Catheter-related infections in neonatal intensive care units: a prospective multicentre surveillance

**DOI:** 10.1186/1753-6561-5-S6-O7

**Published:** 2011-06-29

**Authors:** K Bellemin, N Voirin, M Bonfils, H Bouamari, A Vincent, M-L Valdeyron, B Reygrobellet, P Vanhems, O Claris

**Affiliations:** 1 Hospices Civils De Lyon, Lyon, France

## Introduction / objectives

Most infants in neonatal intensive care units (NICU) are exposed to central venous catheter (CVC) and to central-line-associated bloodstream infections and clinical sepsis (CLABICS). The objectives are to measure incidence and assess risk factors of CLABICS.

## Methods

A prospective surveillance including all neonates with CVC hospitalized in level 3 NICU is ongoing since 2007. Data are collected using standardized forms validated by a referent physician. Incidence rates (IR) with their 95% confidence intervals (95%CI) were calculated as the number of CLABICS divided by the cumulative number of catheter-days at risk. Risk factors were assessed using adjusted hazard ratio (aHR) in Cox regression.

## Results

A total of 1111 neonates exposed to 2049 CVC totalling 14091 catheter-days at risk were included between January 2007 and May 2009. The median gestational age was 30 weeks (range 24-42) and the median birth weight was 1240g (range 440-4400). During study period, 256 CLABICS were detected corresponding to an IR of 16.0 per 1000 catheter-days (95%CI 14.0-18.2): 6.5 (95%CI 4.3-9.3) for umbilical CVC and 20.2 (95%CI 17.5-23.1) for other CVC. The median time to CLABICS was 8 days. Coagulase-negative (82.1%) and aureus (10.8%) staphylococci were the most frequent germs isolated. In multivariate analysis, a birth weight ≤750g (aHR 6.3, 95%CI 1.0-38.1) and intravenous lipid emulsion (aHR 2.3, 95%CI 1.3-3.9) were significantly associated with CLABICS for umbilical CVC. Similar results were observed for other CVC.

## Conclusion

The incidence is high in this cohort especially for non umbilical CVC. Comparing IR and risk factors between NICU may allow adapted control measures to be taken. This surveillance will pursue now into a regional survey.

## Disclosure of interest

None declared.

